# Supplementary material for: Structural basis for LIN54 recognition of CHR elements in cell cycle-regulated promoters
Source: Nat Commun. 2016 Jul 28;7:12301. doi: 10.1038/ncomms12301 (PMC4974476; doi:10.1038/ncomms12301)
Supplement: Supplementary Information — Supplementary Figures 1-10 and Supplementary Table 1. [file ncomms12301-s1.pdf]

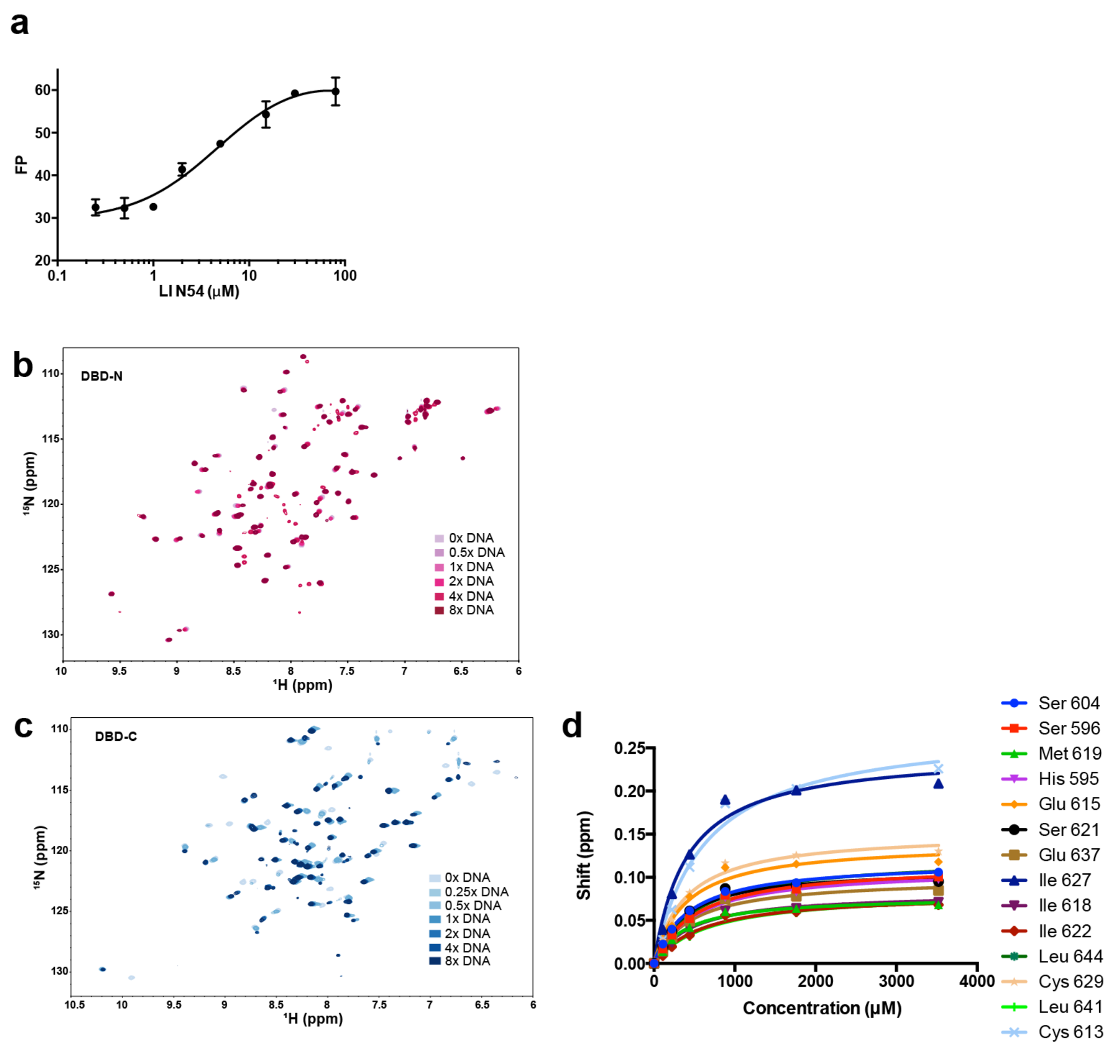

Supplementary Fig. 1. Weak-affinity binding of CHR13 DNA by the DBD-N and DBD-C subdomains. (a) FP assay for CHR13 binding to LIN54 DBD. The measured affinity of CHR13-DBD by FP (K_d = 5 ± 2 μM) is similar to that measured by ITC (K_d = 2.8 ± 0.1 μM) and slightly weaker than the affinity of CHR27 (K_d = 430 ± 80 nM) measured by FP. Error bars show the standard deviation for three experimental replicates. (b,c) HSQC NMR spectra of ^{15}N-labeled 100 μM DBD-N (b) and 440 μM DBD-C (c) in the presence of the indicated concentration of DNA. Peak assignments were completed for DBD-C. (d) Plots of chemical changes as a function of DNA concentration for 14 peaks, corresponding to the indicated residues in DBD-C. Data were fit to a one-site model in GraphPad. We found the average K_d = 500 ± 100 μM. Many peaks in DBD-N shifted only slightly (less than 0.025ppm), others broadened upon addition of DNA.

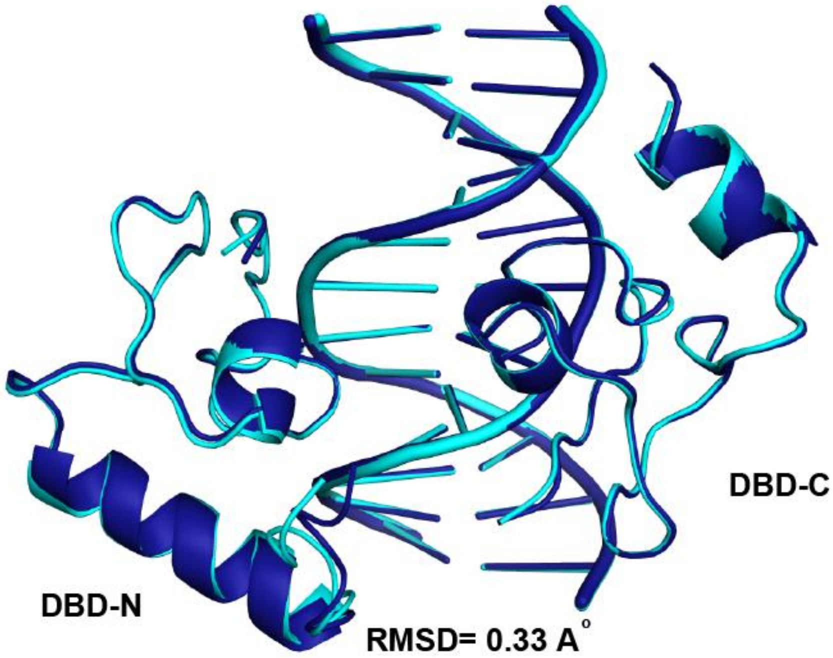

Supplementary Fig. 2. Structural alignment of the two LIN54 DBD-CHR13 complexes in the asymmetric unit. Complexes were aligned using the pairwise algorithm in PyMol. The average C-alpha RMSD = 0.33 Å.

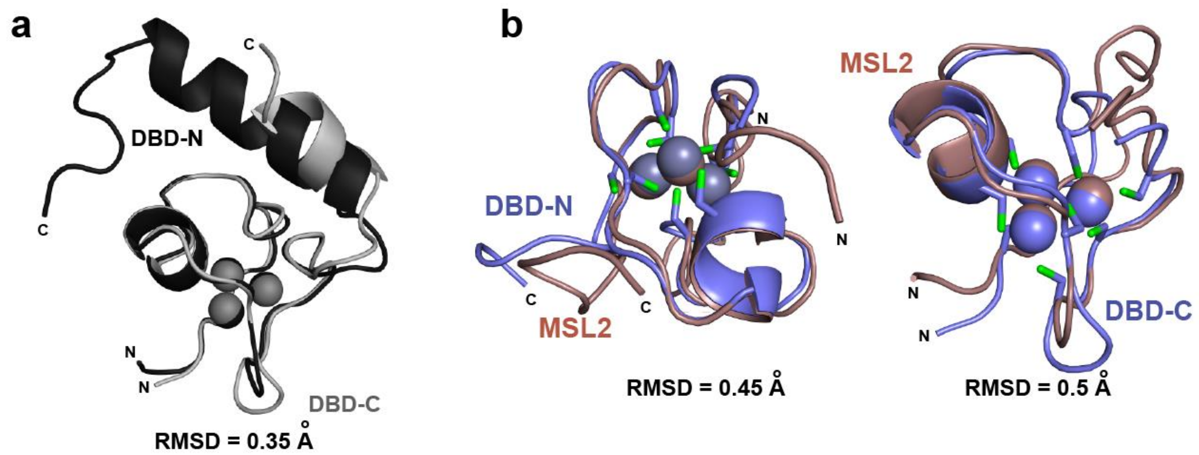

c

```
LIN54 DBD-N  515 -SESASRPRKPCNCTKSL-------CLKLYCDCFANGEFCNN-CNCTNCYNNLEHENERQKAIKACLD
LIN54 DBD-C  589 -GESDRRHSKGCNCKRSG-------CLKNYCECYEAKIMCSSICKCIGCKN-FEESPER-KTLMHLAD
MSL2         291 TVYKKPQEKKGCKCGRATQNPSVLTCRGQRCPCYSNRKACLD-CICRGCQNSYMANGEK--KL
```

Supplementary Fig. 3. Structural alignment of CXC folds from LIN54 and MSL2. (a,b,) The CXC fold from DBD-N (residues 522-581) (black) and DBD-C (597-644) (gray) were aligned with each other (a) and with the CXC domain from MSL2 (residues 522-570 chain A, PDB ID: 4RKH). (b). Structures were aligned using the pairwise algorithm in PyMol. The average RMSDs of the C-alpha alignment are shown in each figure panel. Zinc atoms are shown as grey spheres and the coordinating cysteines are show as sticks. (c) Sequence alignment of the LIN54 CXC folds with MSL2. The tyrosine that inserts into the DNA minor groove is marked with a blue star and is an arginine in MSL2.

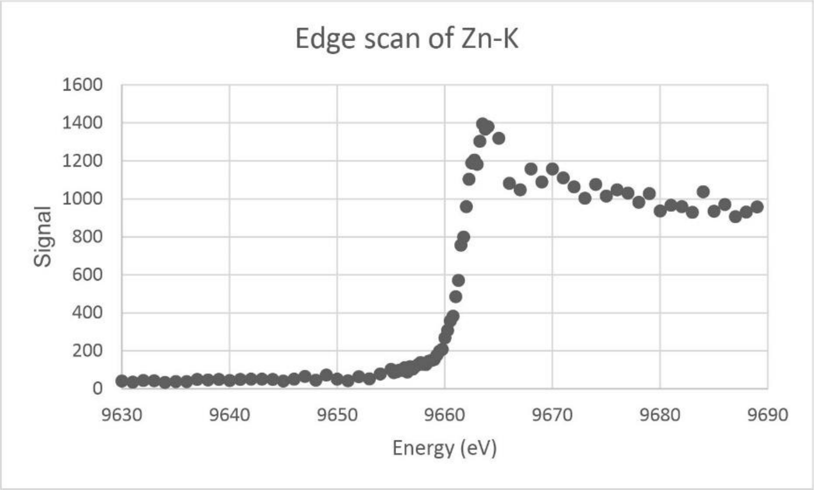

Supplementary Fig. 4. Zn x-ray fluorescence scan.

The ALS 8.3.1 beamline was tuned to the zinc heavy metal wavelength. A DNA-bound LIN54 crystal was scanned for fluorescence at the zinc edge. The signal peak at 9664 eV is consistent with the presence of zinc in the crystal.

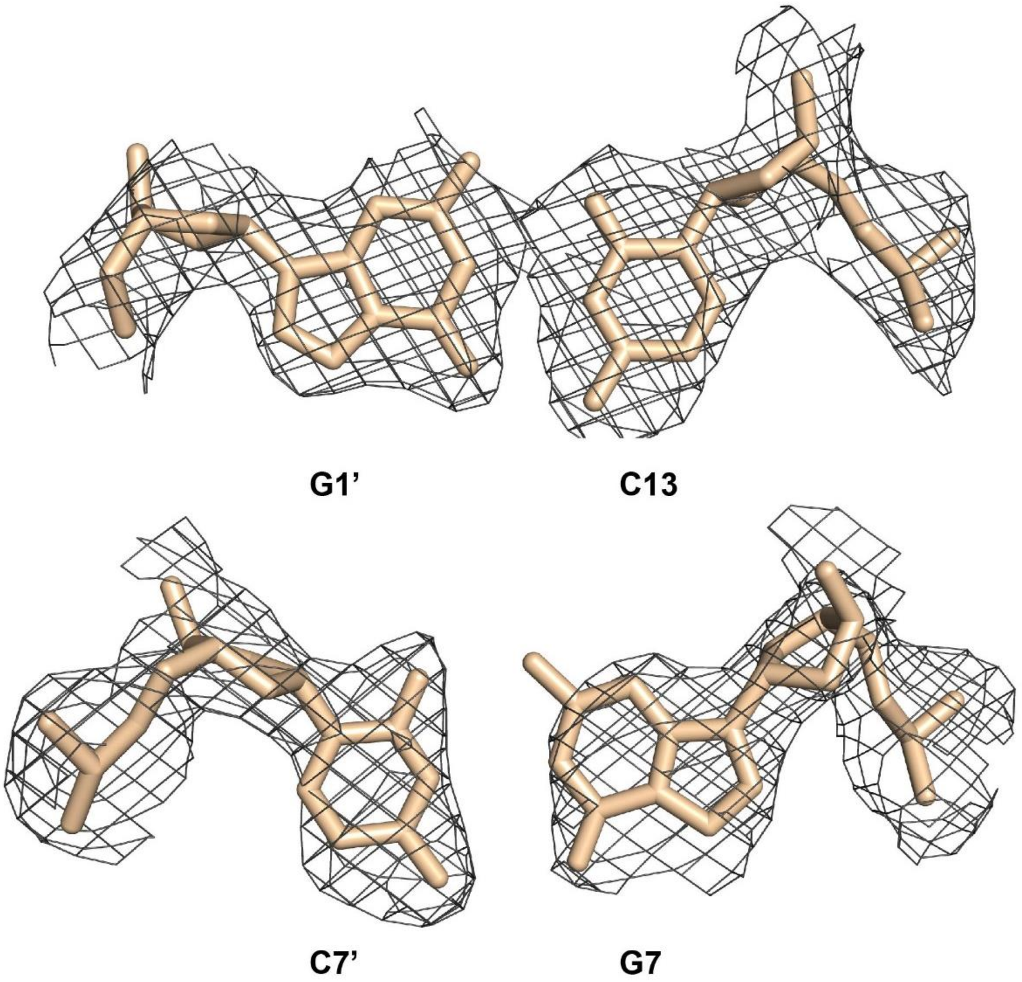

Supplementary Fig. 5. Difference map for G-C and C-G nucleotide pairs. The mesh around each base pair corresponds to a f_o-f_c omit electron density map contoured at 1.8 σ. These particular base pairs are not symmetrical in the DNA molecule and were chosen to highlight unambiguous electron density that defines the directionality of the DNA molecule in the crystal structure.

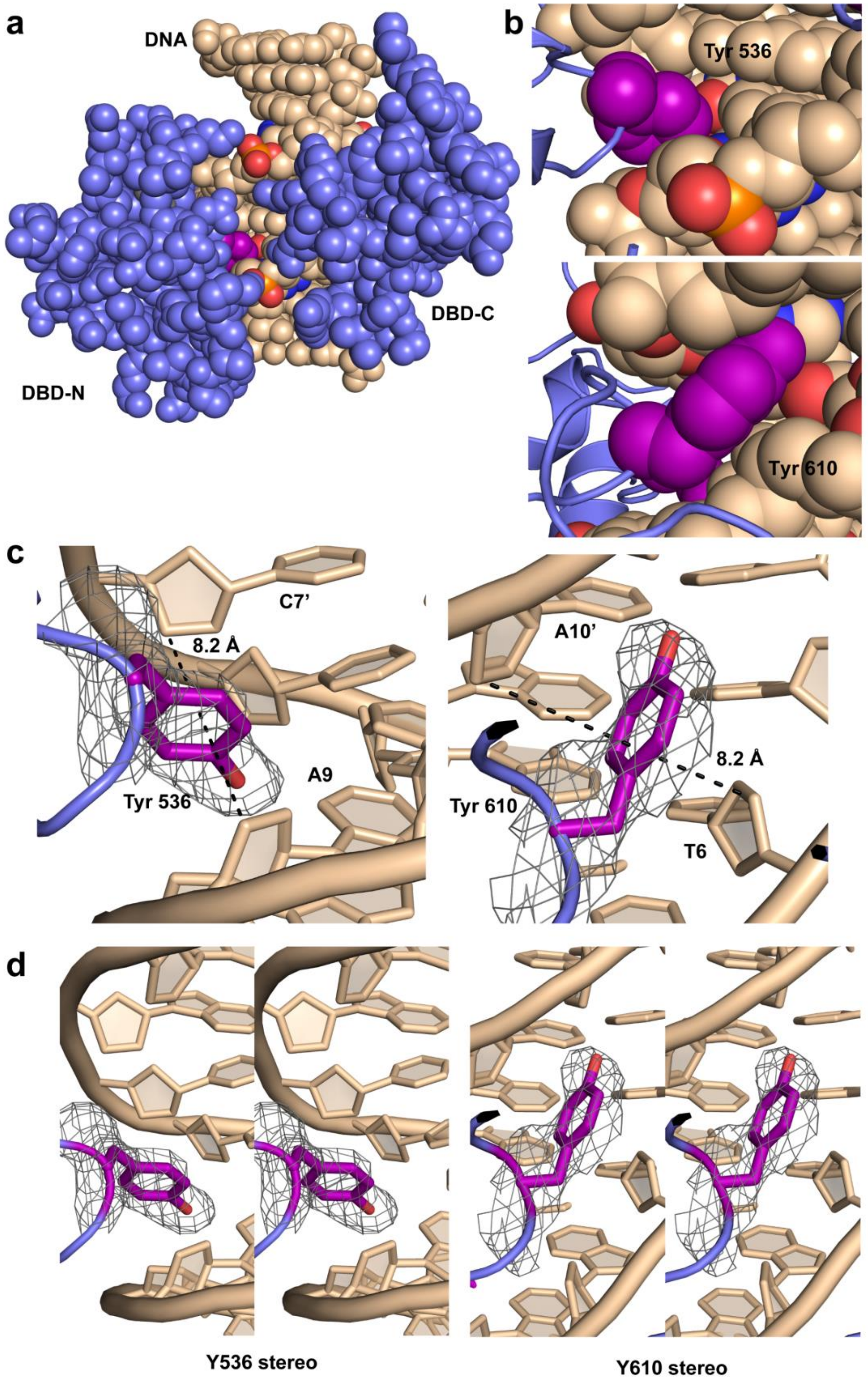

a
DNA
DBD-N
DBD-C
b
Tyr 536
Tyr 610
c
C7'
8.2 Å
Tyr 536
A9
A10'
Tyr 610
8.2 Å
T6
d
Y536 stereo
Y610 stereo

Supplementary Fig. 6. Insertion of LIN54 tyrosines into minor groove. (a,b) Space filling models show binding of the key tyrosine in each LIN54 DBD subdomain. The narrow minor groove optimizes van der Waals contacts. (c) CH-π interactions are between the CH4' of the deoxyriboses on each strand and the tyrosine rings. Vectors are drawn between from CH4' to CH4' to show orientation and distances of the interactions. The mesh corresponds to a f_o-f_c electron density map contoured at 1.6 σ. (d) Stereo images of key tyrosine DNA binding residues from Lin54. The mesh corresponds to a f_o-f_c electron density map contoured at 1.6 σ.

a

Base-pair	Buckle	Prop-Tw	Opening	Tilt
G-C	5.73 (0.26)	-8.53 (-15.15)	-0.35 (-1.31)	0 (0)
A-T	0.66 (-0.04)	-13.78 (-15.13)	-0.27 (-1.88)	-1.88 (-0.01)
G-C	0.52 (0.26)	-11.97 (-15.15)	7.36 (-1.29)	-0.48 (-0.01)
T-A	-5.56 (0.04)	-21.41 (-15.13)	0.58 (-1.87)	0.45 (-0.02)
T-A	-14.02 (0.04)	-21.79 (-15.13)	3.47 (1.88)	0.09 (0)
T-A	-15.44 (0.04)	-9.29 (-15.13)	-3.2 (-1.88)	1.3 (-0.01)
G-C	13.13 (0.26)	-4.62 (-15.15)	1.23 (-1.3)	-0.57 (-0.01)
A-T	15.58 (-0.04)	-24.68 (-15.13)	3.34 (-1.87)	-4.61 (-0.01)
A-T	9.26 (-0.04)	-24.76 (-15.13)	3.31 (-1.87)	2.8 (0)
A-T	1.54 (-0.04)	-21.2 (-15.13)	-3.81 (-1.88)	1.04 (0)
C-G	-2.09 (-0.26)	-13.89 (-15.15)	4.5 (-1.31)	0.36 (0.02)
T-A	-10.79 (0.04)	-9.18 (-15.13)	2.15 (-1.88)	4.88 (0.01)

* Numbers are the values for the CHR13 DNA parameters in the Lin54 structure and in parentheses the values for the ideal B-form parameters for the identical sequence.

b

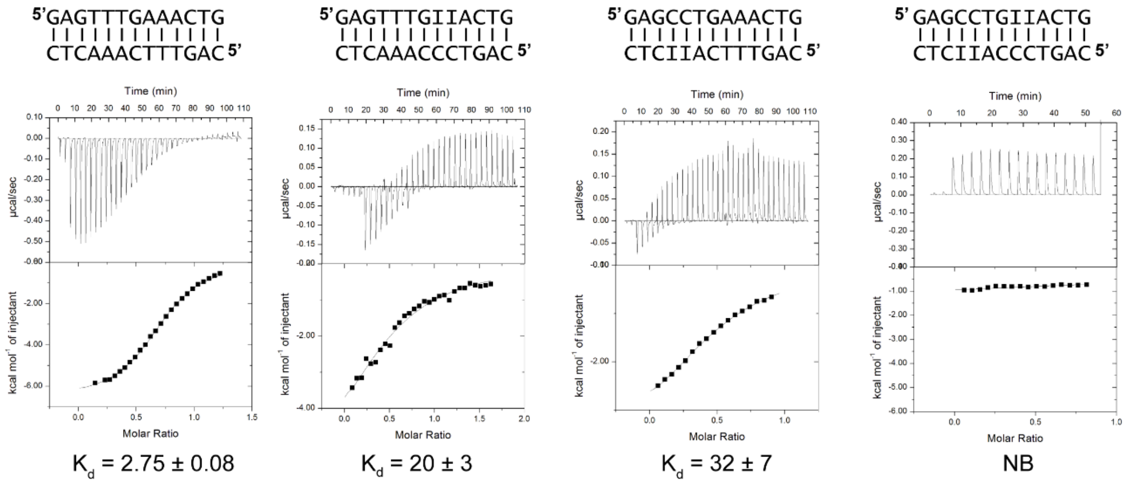

Supplementary Fig. 7. Data supporting preference for the A/T base pairs in the CHR sequence. (a) **Key base and step parameters for the CHR13 DNA.** Numbers are the values for the CHR13 DNA parameters in the LIN54 complex structure and in the parentheses the values for the ideal B-form parameters for the identical sequence. (b) **ITC graphs for the inosine substituted CHR sequences**. The listed sequence show the 13 base duplex DNA used in the ITC binding experiment with LIN54 DBD. The dissociation constants are shown below the binding curves.

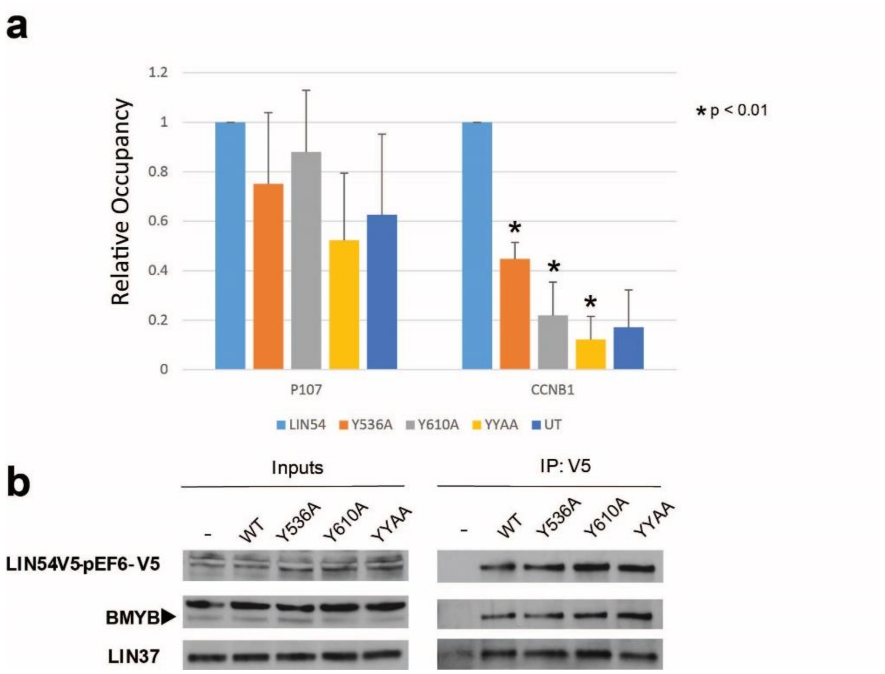

Supplementary Fig 8. Critical DNA-binding tyrosines are necessary for LIN54 recruitment to promoters.

(a) Chromatin immunoprecipitation assay of V5-LIN54 and the indicated mutants after transfection into HeLa cells. Following crosslinking and immunoprecipitation with an anti-V5 antibody, qPCR was performed with primers specific to the *p107* and *CCNB1* promoters. The average promoter enrichment relative to wild-type LIN54 is reported, and the error bars are standard deviations from three biological replicates and one technical replicate. YYAA is the double Y536A and Y610A mutation. The p-value evaluating statistical significance was calculated for the promoter enrichment of a mutant relative to wild-type using a two-tailed student's t-test. There is no significant difference in LIN54, both wild-type and mutant, binding to the noncanonical CHR in the *p107* promoter. (b) Wild-type or mutant LIN54 was transfected into HeLa cells, extracts were immunoprecipitated with an anti-V5 antibody, and precipitates were probed for the indicated proteins. Triangle corresponds to BMYB band in the input samples. All bands shown in the input for Lin54-V5 are non-specific as the protein was undetectable.

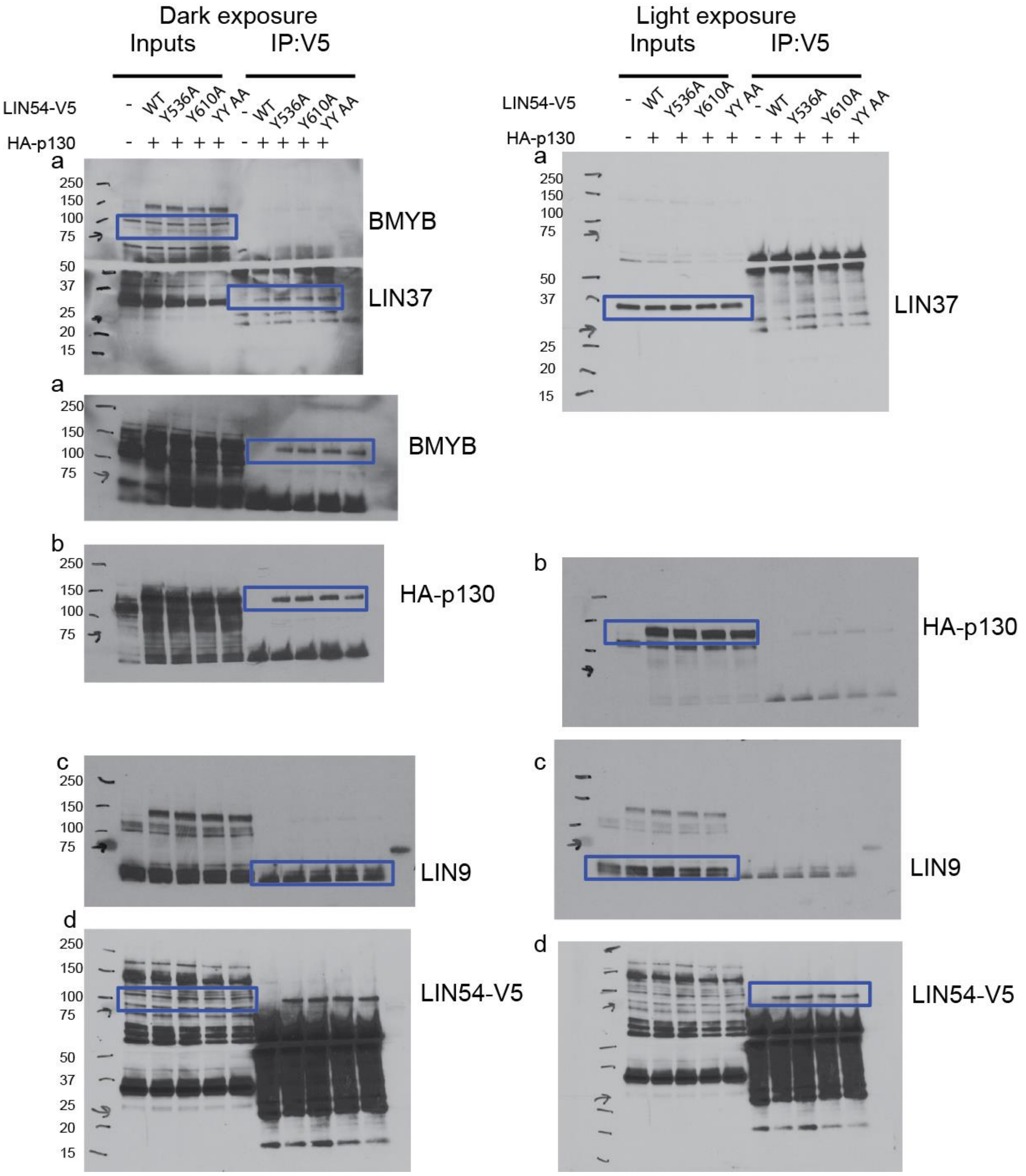

Supplementary Fig 9. Critical DNA-binding tyrosines are necessary for LIN54 recruitment to promoters.

Full western blots for chromatin immunoprecipitation assay of V5-LIN54 and the indicated mutants after transfection into T98G cells, partial blots are shown in Figure 5 in the main text. Two different

exposures are shown for each blot, identical blots with different exposures are indicated by lowercase letters. Blot (a) was cut in half and exposed to either anti-BMYB (higher molecular weight) antibody, or anti-LIN37 (lower molecular weight) as indicated. Boxed bands indicate the area shown in Figure 5. Molecular weight marker is shown on the left most part of the film and the sizes in kilodaltons indicated by the numbers on the left. Blot (b) was exposed to anti-HA antibody, blot (c) was exposed to anti-LIN9 antibody, and blot (d) was exposed to anti-V5 antibody.

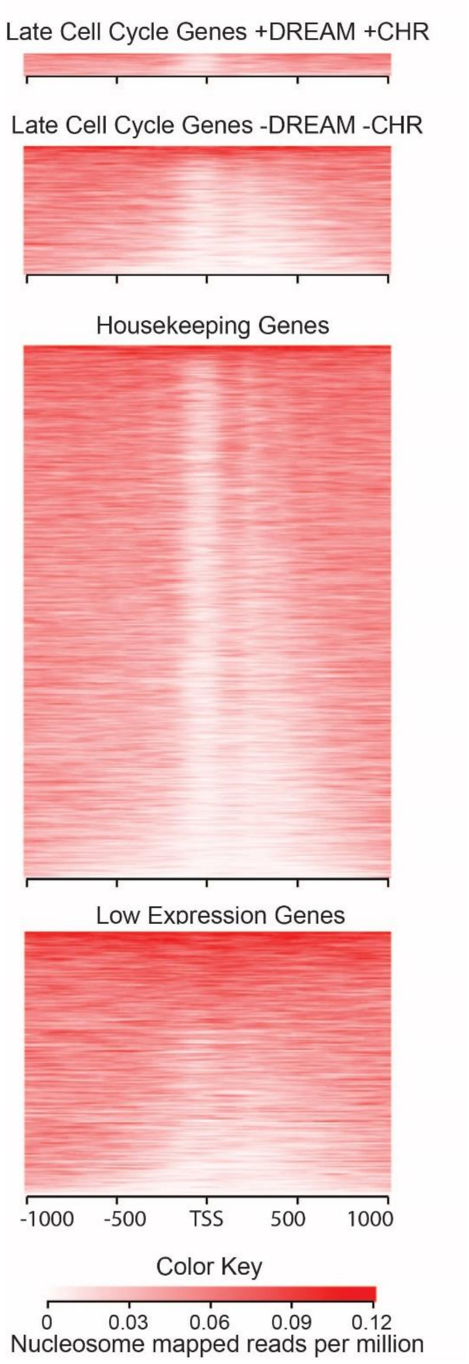

Supplementary Fig. 10. Heatmap of nucleosome positioning. Heatmap of nucleosome positioning data from late cell cycle genes (155) that contain a CHR and confirmed DREAM binding (+DREAM +CHR), late cell cycle genes (899) lacking a CHR and DREAM binding (-DREAM –CHR), (3741) housekeeping genes, and (1732) genes with low levels of expression.

Supplementary Table 1. ITC binding measurements of the LIN54 DBD to the CHR13 DNA sequence.

**Protein**	**Kd (μM)**
LIN54	2.8 ± 0.1
Y536A	No binding
Y536F	No binding
Y536R	No binding
Y610A	No binding
Y610F	No binding
Y610R	No binding
